# Overexpression of MACC1 and Its significance in human Breast Cancer Progression

**DOI:** 10.1186/2045-3701-3-16

**Published:** 2013-03-18

**Authors:** Yongbo Huang, Huizhong Zhang, Junchao Cai, Lishan Fang, Jueheng Wu, Caisheng Ye, Xun Zhu, Mengfeng Li

**Affiliations:** 1 Department of Microbiology, Zhongshan School of Medicine, Sun Yat-Sen University, Guangzhou, Guangdong 510080, China; 2 Key Laboratory of Tropical Disease Control (Sun Yat-Sen University), Ministry of Education, Guangzhou, Guangdong 510080, China; 3 Department of Pathology, Sun Yat-sen University Cancer Center, Sun Yat-Sen University, Guangzhou, Guangdong, China; 4 Department of Breast Disease Center, The First Affiliated Hospital, Sun Yat-Sen University, Guangzhou 510080, China

**Keywords:** Biomarker, Breast cancer, Cancer progression, MACC1, Metastasis

## Abstract

**Background:**

Metastasis-associated in colon cancer-1 (MACC1) was first identified as a transcriptional activator for proto-oncogene c-MET expression, and its overexpression is frequently associated with metastatic progression for multiply tumor types. In the present study, we analyzed for the first time the expression of MACC1 in breast cancer and its correlation with clinicopathologic features, including metastasis and patient survival.

**Results:**

MACC1 protein expression was analyzed in two cohorts of clinicopathologically characterized breast cancer using immunohistochemistry. Statistical analysis showed a significant correlation of MACC1 expression with the primary tumor, lymph node metastasis, distant metastasis classifications as well as the clinical staging in breast cancer patients. Moreover, overexpression of MACC1 was associated with both a reduced recurrence-free survival (RFS) and poorer patients' overall survival (OS). Multivariate analysis with a Cox proportional-hazards model suggested that MACC1 expression was an independent prognostic indicator for RFS and OS. Stratification of breast cancer patients according to the estrogen receptor (ER) status revealed that MACC1 was prognostic for both ER-negative and ER-positive patients.

**Conclusions:**

MACC1 may represent a potentially useful biomarker for the prognosis of breast cancer patients and might be involved in progression of breast cancer.

## Background

Metastasis-associated in colon cancer-1 (MACC1) was first identified to be overexpressed in primary and metastatic tumor specimens of colon cancer as compared to normal colon mucosa and was found to be indicative of metastasis as well as poor survival of the patients. Furthermore, MACC1 has been found to stimulate proliferation, motility and invasion in colon cancer cells through transcriptionally upregulating c-MET [1]. Moreover, studies by other researchers have also linked MACC1 upregulation to cancer development and progression in several types of solid tumors, including lung adenocarcinoma, gastric cancer and hepatocellular carcinoma (HCC) [2-6]. These studies revealed that MACC1 might play a role in the recurrence, metastasis and patient survival in various types of human cancers.

Worldwide, breast cancer is by far the most common cancer in women, and the second most common type of cancer for the general population [7]. Incidence of the disease continues to rise in many countries [8]. Although patient survival for breast cancer has been improved in recent decades [9], the outcome of patients with metastatic breast cancer remains poor with a median overall survival time of 2 to 3 years [10]. It is estimated that 6-10% of breast cancer patients have undergone metastasis at diagnosis [11,12]. Moreover, breast cancer metastasis can occur years or decades after mastectomy [13]. The poor outcome of metastatic breast cancer patients underscores the importance of defining molecular factors responsible for cancer metastasis. Therefore, identifying risk markers to elucidate pathways responsible for breast cancer metastasis is important for the improvement of the risk classification in patients as well as discovery of therapeutic targets.

Breast cancer is a heterogeneous disease with a variety of different molecular subtypes and varied clinical outcomes. To date, molecular classification of breast cancer based on the estrogen receptor (ER), progesterone receptor (PR) and HER2 expression status in clinical setting to differentiate a prognosis and guide treatment. Because of the strong predictive importance of ER to endocrine therapy, breast cancer is generally classified into ER+ and ER- subtypes. Despite existing controversies over the issue, expression of ER is reportedly an important favorable prognostic marker in the first 5 years after diagnosis [14]. On the other side, ER- breast cancers generally have worse outcomes and less treatment options than ER+ disease [15]. However, a subset of ER- breast cancers, such as adenoid cystic carcinoma and secretary carcinoma, are ER-negative and exhibit an optimistic prognosis [16,17]. In this context, biomarkers that can identify individuals with a favorable prognosis among ER- negative breast cancer patients, for whom the toxic chemotherapy might be avoidable, are of value in choosing appropriate adjuvant therapy. Unfortunately, insufficient availability of biomarkers for such a purpose remains a challenge in the clinic.

HER2, a member of the receptor tyrosine kinases (RTK) family that are hyperactive in breast cancer, plays an important role in the initiation and progression of the disease and is associated with poor disease-free and overall survival [18-21]. Clinical trials have shown that treatment with anti-HER2 monoclonal antibodies for breast cancer cases that overexpress HER2 is effective in increasing patient survival [21,22]. However, overexpression of HER2 has been found in only approximately 25% of breast cancer, highlighting the importance of identifying other RTKs involved in the pathogenesis of the disease. One of such potential candidates is c-MET, the product of the proto-oncogene c-*MET*, overexpression of which has been widely reported in breast cancer, and deregulation of c-MET and its ligand, hepatocyte growth factor/scatter factor (HGF/SF), has been found to promote breast cancer progression and correlates with poor survival [23-25] (also visit http://www.vai.org/Met/Index.aspx for a comprehensive list of HGF/SF, c-MET and cancer references). Aberrant activation of the HGF/c-MET pathway can be caused by c-MET mutations, receptor overexpression and amplification, as well as by elevated HGF [26]. In addition, upregulation of MACC1, which controls c-MET transcription, might be a new regulatory mechanism that leads to c-MET activation. However, the expression level of MACC1 in breast cancer and its correlation with the clinical outcome of the disease is unknown. To address this issue, we examined for the first time the expression status of MACC1 in human breast cancer in this study. By using two large cohorts of breast cancer cases, we have found that the level of MACC-1 significantly correlates with the clinical staging and tumor-node-metastasis (TNM) classification of the disease. More importantly, our study also strongly suggests that MACC1 might be an independent biomarker for the prediction of progression and prognosis of breast cancer.

## Results

### MACC1 expression level and Clinicopathologic Characteristics

**Figure 1:**
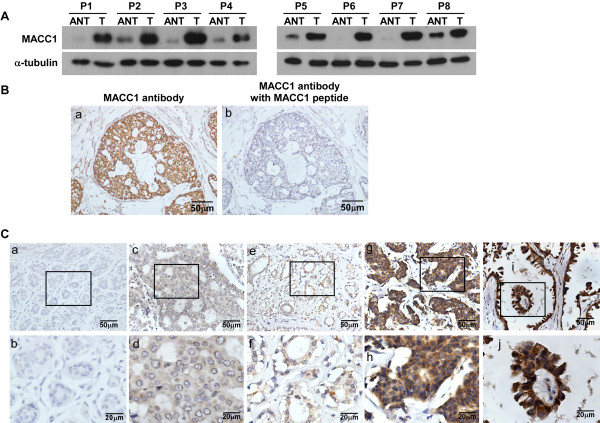
**Expression of MACC1 is elevated in breast cancer. A.** expression of MACC1 protein in each of the primary breast tumors (T) and adjacent normal breast tissue (ANT) samples paired from the same patients by Western blotting. **B.** Validation for the specificity of the antibody against MACC1. Breast cancer sections were immunostained with the MACC1 antibody alone (a) or previously incubated and thereby blocked with a recombinant MACC1 peptide (b). **C.** Representative IHC images of MACC1 expression in normal breast tissue and breast cancer specimens. MACC1 expression was only marginally detectable in normal breast tissues (a and b); (c) and (d)/ (e) and (f), representative images of low MACC1 expression in breast cancer tissues; (g) and (h)/ (i) and (j), representative images of high MACC1expression in breast cancer tissues.

MACC1 expression level was firstly tested by Western blotting analysis in eight paired normal breast tissue and breast tumor specimens from the same patients. As shown in Figurer 1A, protein levels of MACC1 were differentially upregulated in all 8 breast cancer samples as compared with their matched adjacent non-tumor tissue specimens. In order to determine whether MACC1 is clinically correlated with breast cancer progression, the expression of MACC1 was examined by IHC in two large cohorts of breast cancer patients. The clinicopathologic characteristics of the study population are summarized in Table 1. The IHC analysis showed that MACC1 staining was only marginally detectable in normal breast tissue, but in contrast, it was found dramatically overexpressed in breast cancer lesions (Figure 1B and C). In cohorts 1 and 2, 55.5% and 60.0% of patients exhibited high levels of MACC1 expression in their tumor samples, respectively, while, the remaining 44.5% and 40.0% of tumor samples exhibited low expression of MACC1. MACC1 expression was not associated with age, ER and PR status and Her2 status, but was significantly associated with T classification, N classification, distant metastasis status, and clinical stage in both study cohorts (Table 2). Taken as a whole, the expression of MACC1 was positively correlated with clinical staging and TNM classification of breast cancer.

**Table 1:** Clinicopathologic characteristics of patient samples in Cohorts 1 and 2

**Characteristic**	**Cohort 1**		**Cohort 2**	
	**NO.**	**%**	**NO.**	**%**
Total	245		185	
Age				
≤45	95	38.8	78	42.2
>45	150	61.2	107	57.8
Menopausal status				
Premenopausal	116	53.2		
Postmenopausal	102	46.8		
Unknown	27			
Clinical stage				
I	81	33.1	21	11.4
II	91	37.1	80	43.2
III	40	16.3	59	31.9
IV	33	13.5	25	13.5
T classification				
T_1_	110	44.9	36	19.5
T_2_	107	43.7	92	49.7
T_3_	18	7.3	41	22.2
T_4_	10	4.1	16	8.6
N classification				
N_0_	135	55.1	70	37.8
N_1_	51	20.8	78	42.2
N_2_	41	16.7	30	16.2
N_3_	18	7.3	7	3.8
Distant metastasis				
Yes	212	86.5	177	95.7
No	33	13.5	8	4.3
Estrogen receptor				
Positive	174	71.0	101	54.6
Negative	71	29.0	84	45.4
Unknown	0		0	
Progesterone receptor				
Positive	146	66.3	110	59.5
Negative	74	33.7	75	40.5
Unkown	25		0	
Her2				
Positive	43	22.3	21	21.4
Negative	150	77.7	77	78.6
Unknown	52		87	
MACC1 expression				
High expression	136	55.5	111	60.0
Low expression	109	44.5	74	40.0
Endocrine therapy				
tamoxifen	122	43.8		
No treatment	95	56.2		
Unknown	28			

**Table 2:** Correlation between MACC1 expression and the clinicopathologic characteristics of the breast cancer patients

**Characteristics**	**Cohort 1**			**Cohort 2**		
	**MACC1 expression**			**MACC1 expression**		
	**Low**	**High**	***P*-value**	**Low**	**High**	***P*-value**
Age						
≤45	44	51	0.647	29	49	0.504
>45	65	85		45	62	
Menopausal status						
Premenopausal	56	60	0.799			
Postmenopausal	51	51				
Clinical stage						
I	43	38	0.001	19	2	<0.001
II	45	46		37	43	
III	15	25		14	45	
IV	5	28		4	21	
T classification						
T_1_	61	49	0.019	23	13	<0.001
T_2_	39	68		39	53	
T_3_	6	12		11	30	
T_4_	3	7		1	15	
N classification						
N_0_	67	68	0.050	44	26	<0.001
N_1_	25	26		24	54	
N_2_	12	29		4	26	
N_3_	5	13		2	5	
Distant metastasis						
Yes	104	108	<0.001	0	8	0.022
No	5	28		74	103	
Estrogen receptor						
Positive	79	95	0.653	36	65	0.185
Negative	30	41		38	46	
Progesterone receptor						
Positive	63	83	0.990	43	67	0.760
Negative	32	42		31	44	
Her2						
Positive	21	22	0.862	8	13	0.480
Negative	71	79		36	41	
Endocrine therapy						
tamoxifen	62	60	0.614			
No treatment	45	50				

### MACC1 expression level in breast tumor is associated with reduced recurrence-free survival

**Figure 2:**
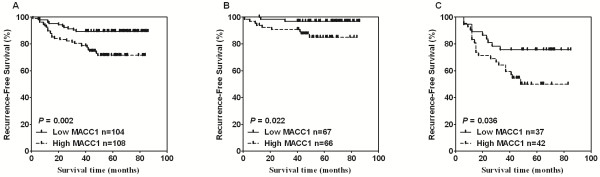
**MACC1 expression in breast tumors is associated with reduced recurrence-free survival. A.** Kaplan-Meier analysis of the probability of cumulative recurrence-free survival in breast cancer cases. **B** and **C**. recurrence-free survival analysis of MACC1 in high- and low-expression groups in LN- (**B**) and LN+ (**C**) subgroups.

The finding that MACC1 overexpression correlated with positive lymph node status and was more commonly present in patients with distant metastasis at diagnosis prompted us to further analyze the association of MACC1 expression with breast cancer recurrence after treatment in cohort 1. Accordingly, patients who developed metastatic disease at diagnosis, as detected and confirmed by computed tomography and/or magnetic resonance imaging and/or positron emission tomography, were excluded from this analysis. Among the 212 cases without any detectable metastases in common metastatic organs at diagnosis, 40 patients (19.0%) relapsed during a median follow-up period of 51 months, and the recurrence-free survival (RFS) was analyzed. Our data showed that the low-MACC1 expression group exhibited a lower rate of recurrence (10.6%) during the follow-up compared with the high-MACC1 group (26.9%, *P* = 0.002). Moreover, Kaplan-Meier analysis and the log-rank test were used to calculate the effect of MACC1 expression on RFS, and the results showed that RFS was significantly different between the low- and high-MACC1 expression groups (*P* = 0.002, Figure 2A), strongly indicating that MACC1 overexpression generally correlated with breast cancer recurrence. As breast cancer patients with positive lymph nodes (LN+) are far more likely to relapse than those with negative lymph nodes (LN-) [27], in order to investigate the prognostic value of MACC1 in LN- and LN + patients, LN status stratification were employed for further Kaplan-Meier analysis on correlations of RFS with the MACC1 expression level in these two groups. The analysis found that the MACC1 expression is significantly associated with RFS of patients in both LN-negative and positive groups (Figure 2B and C). Furthermore, a multivariate Cox regression analysis showed that after adjustment of tumor size and lymph node status, MACC1 was an independent prognostic factor for RFS in breast cancer (*P* = 0.006, Hazard ratio: 2.378, 95% CI, and 1.279 to 4.424, Table 3). Collectively, our results revealed that high MACC1 expression not only was more commonly found in patients with LN or distant organ metastasis, but also represented an independent prognosis marker for RFS.

**Table 3:** Univariate and multivariate analyses of various prognostic variables for recurrence-free survival in cohort 1

	**Univariate analysis**		**Multivariate analysis**	
	***P*-value**	**Hazard ratio (95% CI)**	***P*-value**	**Hazard ratio (95% CI)**
Age				
≤45	0.202	0.719 (0.433-1.193)		
>45				
Menopausal status	0.768	0.915 (0.508-1.648)		
Premenopausal				
Postmenopausal				
Tumor size (cm)				
≤2	0.000	3.479 (1.881-6.435)	0.146	1.649(0.840-3.237)
>2				
Lymph node status				
Negative	<0.001	5.424 (2.932-10.036)	<0.001	4.205(2.205-8.017)
Positive				
ER				
Positive	0.012	0.519 (0.310-0.866)	0.060	0.606(0.359-1.021)
Negative				
PR				
Positive	0.020	0.528 (0.308-0.906)		
Negative				
ErbB-2				
Positive	0.220	1.527 (0.777-3.004)		
Negative				
MACC1 expression				
Positive	0.000	3.021 (1.659-5.501)	0.006	2.378(1.279-4.424)
Negative				

### MACC1 expression and overall survival analysis in test and verification cohorts

**Figure 3:**
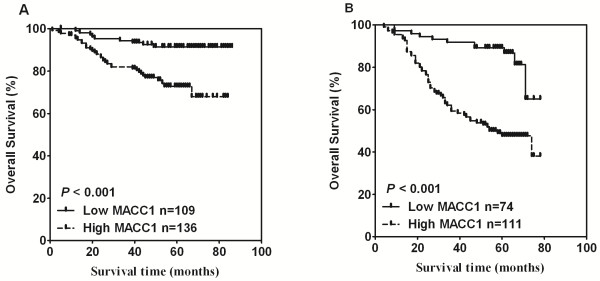
**MACC1 expression and overall survival analysis. A**. Kaplan-Meier curves with univariate analysis (log-rank) for patients with low- versus high-MACC1 expression tumors in cohort 1. **B**. Overall survival analysis and comparison in patients with high- and low-MACC1 groups in cohort 2.

We next examined the effectiveness of MACC1 expression in predicting overall survival of breast cancer patients. Kaplan-Meier analysis and log-rank test were used to calculate the effects of MACC1 expression on overall survival. The log-rank test showed that overall survival was significantly different between the low- and high-MACC1 expression groups (*P* <0.001, Figure 3A). Specifically, the cumulative 5-year survival rate was 91.44% (95% confidence interval, 0.841 to 0.954) in the low-MACC1 expression group (n = 109), as opposed to the 72.78% survival rate (95% confidence interval, 0.634 to 0.800) in the high-MACC1 expression group (n = 136). Furthermore, univariate and multivariate analyses were performed, and as shown in Table 4, a multivariate Cox regression analysis showed that after adjustment of tumor size, lymph node status and human hormone receptor status, MACC1 was an independent prognostic factor for overall survival in breast cancer. (*P* = 0.010, Hazard ratio: 2.730, 95% CI, 1.277 to 5.839, Table 4). To confirm our finding in the cohort 1, an independent second cohort of 185 patients from a different hospital, the Sun Yat-Sen University Cancer Center, was used for further analysis. Patients in cohort 2 were again classified into high- and low-MACC1 expression groups using an identical IHC method. We found that patients with high MACC1 expression had poorer overall survival than patients with low MACC1 expression (*P* < 0.001, Figure 3B). Likewise, MACC1 was an independent prognostic factor for overall survival in breast cancer when a multivariate Cox regression analysis was used in cohort 2 (*P* = 0.001, Hazard ratio: 3.190, 95% CI, 1.651 to 6.163, Table 5). Taken together, our results suggest that MACC1 might represent a novel and potentially useful independent biomarker for the prognosis of patients with breast cancer.

**Table 4:** Univariate and multivariate analyses of various prognostic variables for overall survival in cohort 1

	**Univariate analysis**		**Multivariate**	
	***P***	**Hazard ratio (95% CI)**	***P***	**Hazard ratio (95% CI)**
Age				
≤45	0.261	0.709 (0.389-1.291)		
>45				
Menopausal status	0.589	1.223 (0.590-2.534)		
Premenopausal				
Postmenopausal				
Tumor size (cm)				
≤2	<0.001	5.930 (2.501-14.064)	0.027	2.833(1.125-7.132)
>2				
Lymph node status				
Negative	<0.001	5.581 (2.674-11.648)	0.001	3.836(1.786-8.239)
Positive				
ER				
Positive	0.11	0.458 (0.251-0.836)	0.044	0.534 (0.290-0.983)
Negative				
PR				
Positive	0.076	0.557 (0.292-1.064)		
Negative				
ErbB-2				
Positive	0.837	1.101 (0.440-2.758)		
Negative				
MACC1 expression				
Positive	0.001	3.682 (1.756-7.721)	0.010	2.730(1.277-5.839)
Negative				

**Table 5:** Univariate and multivariate analyses of various prognostic variables for overall survival in cohort 2

	**Univariate analysis**		**Multivariate**	
	***P***	**Hazard ratio (95% CI)**	***P***	**Hazard ratio (95% CI)**
Age				
≤45	0.808	1.061 (0.657-1.713)		
>45				
Tumor size (cm)				
≤2	<0.001	3.449 (2.131-5.584)	<0.001	2.834(1.741-4.612)
>2				
Lymph node status				
Negative	<0.001	3.657 (1.960-6.823)	0.001	2.936(1.540-5.597)
Positive				
ER				
Positive	0.793	0.938 (0.583-1.509)		
Negative				
PR				
Positive	0.036	0.603 (0.375-0.968)	0.002	0.465 (0.286-0.757)
Negative				
ErbB-2				
Positive	0.886	0.937 (0.383-2.292)		
Negative				
MACC1 expression				
Positive	<0.001	4.564 (2.392-8.709)	0.001	3.190 (1.651-6.163)
Negative				

### MACC1 overexpression identifies patients with poor clinical outcome within ER-positive or -negative subgroups of breast cancer

**Figure 4:**
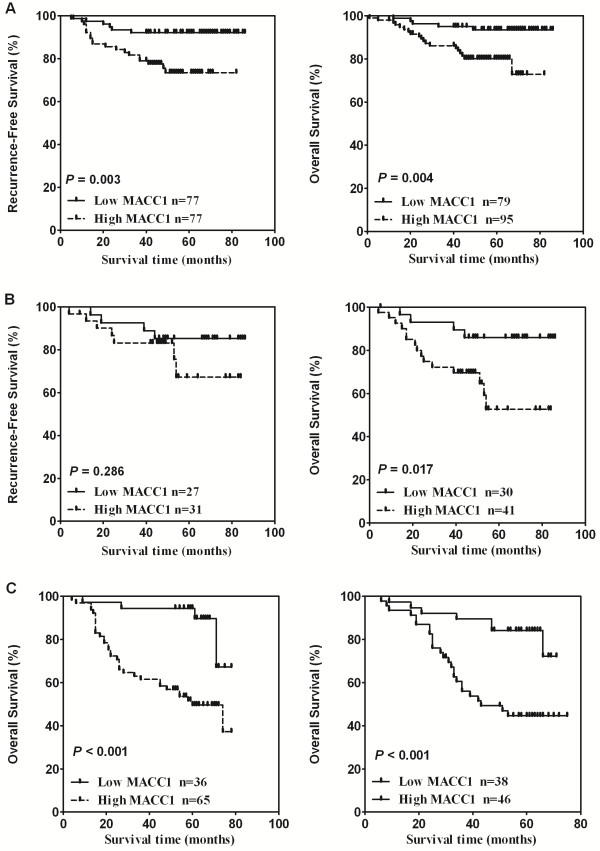
**Kaplan-Meier estimates of relapse-free survival and overall survival according to MACC1 expression level in subgroups of ER- and ER+ patients in two independent datasets. A**. Relapse-free survival and overall survival of ER+ breast cancer patients in cohort 1. **B**. Relapse-free survival and overall survival of ER- breast cancer patients in cohort 1. **C**. Overall survival of ER+ and ER- breast patients in cohort 2.

Because ER-positive and -negative breast cancers are considered to be two clinically as well as biologically different disease entities, we then separately investigated the prognostic value of MACC1 expression in these tumor subtypes. We stratified these patients into ER-positive and ER-negative groups according to the proportion of positive ER stain cells (cutoff: ≥10%) and conducted Kaplan-Meier analysis survival analysis and long-rank test for each subgroup. Among the 154 breast cancer cases with positive ER status in cohort 1, high expression MACC1 was significantly associated with relapse-free survival (*P* = 0.003, Figure 4A, left). For the overall survival, there was a significant difference between the MACC1 high- and low-expression groups (Figure 4A, right, *P* = 0.004). We also conducted an overall survival analysis in 101 patients with ER-positive tumors in validation cohort 2, and the association of high expression of MACC1 with poor prognosis was confirmed (Figure 4C, left, *P* < 0.001).

Among ER-negative cases, the Kaplan-Meier analysis showed that MACC1 expression can predict survival in these patients. High MACC1 expression was significantly associated with decreased overall survival of these patients both in cohort 1 and cohort 2 (*P* =0.017 Figure 4B, right and *P* <0.001, Figure 4C, right, respectively). For the relapse-free survival curves shown in ER- breast cancer patients, although the survival curve for MACC1 high-expression patients lied marginally below that for the MACC1 low-expression group, the difference was not statistically significant (*P* = 0.286). This may be due to the relatively small size of the study population (n = 58), and apparently further investigation is needed for a definite conclusion.

Finally, to increase the power of the analysis, we pooled two prognostic datasets and examined whether the MACC1 was an independent prognostic factor for overall survival in ER+ as well as ER- breast cancer patients. The results showed that the prognostic value of MACC1 level in predicting poor survival in ER-positive and -negative breast cancers, respectively, remains significant in the multivariate analysis (*P* < 0.001, Hazard ratio:3.747, 95% CI, 1.799 to 7.805 and *P* = 0.001, Hazard ratio:3.076, 95% CI, 1.560 to 6.064, Table 6).

**Table 6:** Multivariate analysis of various prognostic variables for overall survival in ER+ and ER- breast cancer subtypes

	**ER+**		**ER-**	
	***P***	**Hazard ratio (95% CI)**	***P***	**Hazard ratio (95% CI)**
Tumor size (cm)				
≤2	0.004	2.240 (1.289-3.892)	0.070	1.696(0.958-3.000)
>2				
Lymph node status				
Negative	<0.001	3.679 (1.824-7.422)	<0.001	3.671(1.878-7.173)
Positive				
MACC1 expression				
Positive	<0.001	3.747 (1.799-7.805)	0.001	3.076(1.560-6.064)
Negative				

## Discussion

The current study represents the first demonstration of an association between MACC1 expression and breast cancer survival and the value of MACC1 as a prognostic marker for the disease. We find that high MACC1 expression is significantly related to reduced RFS and overall survival of breast cancer patients. As indicated by multivariate Cox regression analysis, MACC1 expression level represents an independent prognostic factor for overall survival. Furthermore, overexpression of MACC1 could identify poorer overall survival within both ER-positive and ER-negative breast cancer patient subgroups.

Metastasis remains the most important cause of deaths in breast cancer, and thus, understanding the clinical significance of metastasis-related molecules is key to developing novel and effective management strategies for breast cancer patients. While in other cancer types, such as colon cancer, gastric cancer and HCC, MACC1 has been found in fair numbers of patients (n = 41 ~ 60) to be pro-metastatic and predictive of disease prognosis [2,3,6], the clinical significance of MACC1 in breast cancer was not previously studied, and whether it is of prognostic value in breast cancer, particularly for different patient subgroups, required enrollment of sufficient number of clinical cases to reach clinically relevant and meaningful conclusion. In our current study, two large independent patient cohorts were employed, with which not only was overexpression of MACC1 in breast cancer identified and validated, but also its correlations with clinical staging, distant metastasis and patient survival were established. It is of particular note that for our cohort 1, in which RFS information was available for the enrolled patients, the study clearly demonstrated that when compared to the MACC1 low-expression group, MACC1 high-expression group showed a 2.5-fold increase of relapse rate. Interestingly, while the expression level of MACC1 correlates with the N classification in breast cancer, it is also of prognostic value in both LN-positive and -negative patients. Importantly, since multivariate analyses suggest that either expression level of MACC1 or lymph node status is an independent prognostic parameter for RFS, quantification of MACC1 expression may represent a useful approach, in addition to lymph node status, to evaluating the risk of recurrence, aside from its identified value for predicting overall survival of breast cancer patients by this study.

The differential clinical manifestations between ER-positive and -negative breast cancers have been recognized for many years [28], and more recently, they are considered to be two different molecular types of the disease characterized by distinct gene expression patterns [29]. Prognostic significance of biomarkers has been found to be inconsistent between ER-positive and -negative breast cancer [30,31]. In previous reports studying the prognosis of the two breast cancer groups, results obtained have been contradictory, possibly reflecting the molecular heterogeneity of breast cancers [32]. In this study, survival analysis was conducted and found to be prognostic in both ER-positive and ER-negative groups in two independent cohorts, it would be of great interest to further explore whether MACC1 plays an important role in promoting tumor progression through mechanisms common in different types of breast cancer. It is well recognized that breast cancer patients with positive ER, which account for at least two-thirds of all breast cancer cases, display better clinical outcomes than those with negative ER. ER-positive breast cancer patients usually benefit from adjuvant endocrine therapy, such as tamoxifen and its analogues [33]. Furthermore, it has been reported that in ER-positive patients, postoperative tamoxifen treatment could highly effectively reduce the chance of recurrence without addition of chemotherapy [34]. However, in the current clinical practice, postoperative chemotherapy is prescribed quite often in many areas of the world, which might subject patients to overtreatment and the accompanying adverse effects. Therefore, it would be clinically important to identify individuals with low-risk of recurrence in ER-positive patients so that unnecessary treatment can be avoided for a significant fraction of breast cancer patients. On the other hand, it is equally important to distinguish favorable prognosis factors for patients bearing ER-negative tumors with good prognosis who may not need further treatment after complete resection. The finding that MACC1 expression has prognostic values in both ER-positive and -negative breast cancer subgroups warrants further evaluation to explore whether the biomarker can be clinically useful in the decision-making process for the treatment of breast cancer patients.

The molecular pathways underlying a possible biological role of MACC1 in breast cancer progression are yet to be elucidated. Previous studies have shown that MACC1 acts as a transcription activator for c-MET and as a key regulator of HGF-c-MET signaling pathway in colon cancer, promoting proliferation, invasion and HGF-induced scattering of colon cancer cells and xenograft growth and metastasis in vivo [1]. Moreover, it has been recognized that the *c-MET* oncogene, which codes for the tyrosine kinase receptor for HGF, enhances invasion and metastasis of various types of cancer cells, including breast cancer, through stimulating proliferation, survival, invasiveness and angiogenesis [35-37]. Furthermore, c-MET overexpression in breast tumors has been found to be associated with disease progression and correlate with poor survival [23,37-41]. Hence, our finding that MACC1 overexpression is closely related to the clinical outcome of breast cancer warrants further and in-depth investigation on whether and how MACC1 interacts with the HGF-c-MET pathway, or other signaling pathways, in this highly prevalent malignancy.

## Conclusions

In conclusion, this is the first study aimed at evaluating the possibility of using MACC1 as a clinically relevant indicator for breast cancer recurrence and as a prognostic marker for patient survival in breast cancer. In addition, MACC1 expression status may be useful for evaluating the effectiveness of novel anti-breast cancer therapeutic strategies and for developing rational criteria for the selection of treatments. Toward this end, further investigation on the mechanism by which MACC1 is involved in the development and progression of breast cancer and prospective studies on the prognostic significance of MACC1 are needed.

## Methods

### Patient information and tissue specimens

This study was conducted on a total of 430 paraffin-embedded breast cancer samples, which were histopathologically and clinically diagnosed at the First Affiliated Hospital of Sun Yat-sen University and the Sun Yat-sen University Cancer Center from 2000 to 2002. In the first cohort, a total of 245 primary breast cancer patients were treated at First Affiliated Hospital of Sun Yat-sen University. Clinical information, including age, tumor size, lymph node status, clinical stage, ER status, PR status, HER2 expression and clinical follow-up data, was obtained from all patients' medical records (when available). For each patient, recurrence-free survival (RFS) was defined as the time interval between the date of diagnosis to first recurrence (including local or regional recurrence and organs metastasis). Overall survival (OS) was defined as the time from date of diagnosis to death whereby breast cancer was the primary or underlying cause of death. Patients who were alive at the last follow-up were censored at the follow-up dates, and patients who died from causes other than breast cancer were censored at the time of death. For the use of these clinical materials for research purposes, prior patients’ consents and approval from the Sun Yat-sen University and First Affiliated Hospital Institutional Board were obtained. All samples were collected and analyzed with prior written informed consent from the patients.

To further verify the results from the first cohort, an independent second cohort of patients was included in this study, which enrolled 185 patients treated at the Sun Yat-Sen University Cancer Center. In the second cohort, only OS information is available as the follow-up data. Clinical and clinicopathologic classification and staging were determined according to the American Joint Committee on Cancer guideline [42]. Clinical information of the patients and samples is summarized in Table 1. For the use of clinical materials for research purposes, prior patients’ consents and approval were obtained from the Sun Yat-Sen University and Cancer Center Institutional Board. All samples were collected and analyzed with prior written informed consent from the patients. Four normal breast tissues were obtained from the reduction mammoplasty material at the Department of Plastic Surgery, the First Affiliated Hospital of Sun Yat-Sen University and approved by the Sun Yat-Sen University and First Affiliated Hospital Institutional Board. Samples were collected and analyzed with written informed consent.

Eight pared tumors and corresponding normal breast tissues were collected at surgically resected tissues at the Sun Yat-Sen University Cancer Center and stored immediately at −80°C until analysis. All specimens were confirmed by routine histopathologic analysis. Written informed consent was obtained from all patients.

### Western blotting

Western blotting analysis was performed as described [43]. Fresh tissue was grounded to powder in liquid nitrogen and then lysed with sampling buffer [62.5 mmol/L Tris–HCl (pH 6.8), 10% glycerol, and 2% SDS] and boiled for 5 min. Protein concentration was determined using the Bradford assay (Bio-Rad Laboratories, Hercules, CA). Equal amounts of protein was separated electrophoretically on 7.5% SDS-polyacrylamide gels and transferred onto polyvinylidene difluoride membranes (Roche Diagnostics, Mannheim, Germany). The membrane was probed with an anti-MACC1 rabbit antibody (1:1,000 dilution; ProSci Inc. Poway, CA). Expression of MACC1 was determined using horseradish peroxidase-conjugated anti-rabbit immunoglobulin G diluted at 1:3000 and enhanced chemiluminescence method (Pierce, Rockford, IL) according to the manufacturer’s suggested protocol. The membrane was stripped and re-probed with an anti-α-tubulin mouse monoclonal antibody (1:1,000 dilution; Sigma-Aldrich, St Louis, MO) as a loading control.

### Immunohistochemistry analysis (IHC)

IHC was performed to investigate altered protein expression in 430 human breast cancer tissues and 4 normal breast tissues. Rabbit anti–MACC1 (1:500; ProSci Inc. Poway, CA) and a MACC1 peptide (ProSci Inc. Poway, CA) was used in this study. The IHC procedure was carried out as previously reported [44].

The degree of immunostaining of formalin-fixed, paraffin-embedded sections was reviewed and scored independently by two observers, who were blinded to clinical data, based on both the proportion of positively stained tumor cells and the intensity of staining [45-47]. The proportion of tumor cells was scored as follows: 0 (no positive tumor cells), 1 (<10% positive tumor cells), 2 (10-50% positive tumor cells), and 3 (>50% positive tumor cells). The intensity of staining was graded according to the following criteria: 0 (no staining); 1 (weak staining = light yellow), 2 (moderate staining = yellow brown), and 3 (strong staining = brown). The staining index was calculated by multiplying the staining intensity score and the proportion of positive tumor cells. Using this method of assessment, we evaluated the expression of MACC1 in benign breast epithelium and malignant lesions by determining the staining index, resulting in scores as 0, 1, 2, 3, 4, 6 or 9. Cutoff values for MACC1 were chosen on the basis of a measurement of heterogeneity with the log-rank test statistical analysis with respect to overall survival. An optimal cutoff value was identified: the staining index score of ≥6 defined tumors as high MACC1 expression and ≤4 as low expression of MACC1. This IHC analysis has been used in previous publications as a widely accepted method [44,48-53].

### Statistical analysis

All statistical analyses were carried out using the SPSS 10.0 statistical software package (SPSS Inc, Chicago, IL). Pearson’s chi-square test was used to analyze the correlation between MACC1 expression and the clinicopathologic characteristics. Survival curves were plotted using the Kaplan-Meier method and compared with the log-rank test. The significance of various variables for survival was analyzed by the Cox proportional hazards model in the multivariate analysis. *P* < 0.05 in all cases was considered statistically significant.

## Abbreviations

ER: Estrogen receptor; HCC: Hepatocellular carcinoma; IHC: Immunohistochemistry; LN: Lymph nodes; MACC1: Metastasis-associated in colon cancer-1; OS: Overall survival; PR: Progesterone receptor; TNM: Tumor-node-metastasis; RFS: Reduced recurrence-free survival.

## Competing interests

The authors declare that they have no competing interests.

## Authors’ contributions

YH, HZ, JC and LF carried out the IHC analysis and statistical analysis. HZ, CY, JW and XZ contributed clinical information. YH, HZ and ML participated in the interpretation of data and the critical review of the manuscript. YH wrote the article and ML revised it. ML provided the general ideas of this manuscript and co-designed and coordinated the study. All authors read and approved the final manuscript.
